# Directing Transition of Synthetic Protocell Models via Physicochemical Cues‐Triggered Interfacial Dynamic Covalent Chemistry

**DOI:** 10.1002/advs.202101187

**Published:** 2021-07-28

**Authors:** Yanglimin Ji, Wenjing Mu, Hua Wu, Yan Qiao

**Affiliations:** ^1^ Beijing National Laboratory for Molecular Sciences (BNLMS) Laboratory of Polymer Physics and Chemistry CAS Research/Education Center for Excellence in Molecular Sciences Institute of Chemistry Chinese Academy of Sciences Beijing 100190 China; ^2^ University of Chinese Academy of Sciences Beijing 100049 China

**Keywords:** compartmentalization, dynamic covalent chemistry, protocell, self‐assembly, synthetic cell

## Abstract

As the preliminary synthetic analogs of living cells, protocells with life‐like features serve as a versatile platform to explore the origin of life. Although protocells constructed from multiple components have been developed, the transition of primitive cellular compartments toward structural complexity and advanced function remains a scientific challenge. Herein, a programmable pathway is established to exploit a simple chemistry to construct structural transition of protocell models from emulsion droplets, nanocapsules to molecularly crowded droplets. The transitional process toward distinct cell‐like compartments is driven by interfacial self‐assembly of simple components and regulated by physicochemical cues (e.g., mechanical force, solvent evaporation, acid/base equilibrium) triggered dynamic covalent chemistry. These protocell models are further studied by comparing their compartmentalization behavior, sequestration efficiency, and the ability to enrich biomolecules (e.g., enzyme and substrate) toward catalytic reaction or biological activity within the compartments. The results showcase physiochemical cues‐driven programmable transition of life‐like compartments toward functionalization, and offer a new step toward the design of living soft materials.

## Graphical abstract

Programmable transitional protocell models achieved by dynamic covalent chemistry are developed. The reversible formation and breakage of dynamic covalent bonds directed the phase transition toward redistribution of hydrophobic and hydrophilic regions. The structural transition feature makes it possible to encapsulate biomolecules, and further promote highly efficient reactions within these cell‐like compartments.

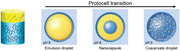

## 1 Introduction

Protocells are rudimentary cell‐like colloidal entities possessing biomimetic functions. Recent years have witnessed rapid advances in the construction of protocell models, which offers a key step to unravel the evolutionary implications for the origin of life and promote the development of synthetic biology.^[^ ^1^, ^2^, ^3^ ^]^ Particular research efforts have been devoted to understanding the formation of membrane‐bound^[^ ^4^, ^5^, ^6^, ^7^, ^8^ ^]^ or membraneless^[^ ^9^, ^10^, ^11^, ^12^ ^]^ colloidal compartments in order to develop functionalized systems with life‐like behaviors, such as compartmentalization,^[^ ^13^, ^14^, ^15^, ^16^, ^17^ ^]^ metabolism,^[^ ^18^, ^19^, ^20^ ^]^ migration,^[^ ^21^, ^22^ ^]^ reproduction,^[^ ^23^, ^24^, ^25^ ^]^ and protocell communication.^[^ ^26^, ^27^, ^28^ ^]^ Notwithstanding the tremendous progress in fabricating synthetic protocells, it remains elusive to explore the possible pathway to direct the transformation of cell‐like compartments into higher‐ordered protocells with spatial compartmentalization and complex function. Previously, protocell transition has been reported in systems such as fatty acid, surfactant/phospholipid and RNA/protein mixtures, in which primitive fatty acid vesicles evolved into phospholipid mixed vesicles, and coacervate droplets transformed into bilayered vesicles.^[^ ^29^, ^30^, ^31^ ^]^

Inspired by this pioneering work, we are seeking for possible pathways of directing the transition of cell‐like compartments using basic chemistry and simple components. To this end, we applied a combination of physiochemical cues (e.g., mechanical force, heating/cooling cycle, acidic/basic condition) to initiate a series of compartmentalization process from a simple biphasic system. Key to this strategy is the utilization of dynamic covalent chemistry (DCC) at oil/water interface to drive molecular assembly into cell‐like objects with distinct compartmentalization. Aligned with supramolecular chemistry, DCC offers new opportunities to construct soft dynamic materials with appealing properties such as stimuli‐responsiveness, self‐healing, and adaptivity, by reversibly break and re‐form chemical bonds^[^ ^32^, ^33^, ^34^, ^35^, ^36^ ^]^ in the presence of stimuli or catalysts (e.g., pH, heat, oxidants, and reductants).^[^ ^37^, ^38^ ^]^ We synthesized a supra‐amphiphile through aldehyde‐amine bond at oil/water interface, which simultaneously generated emulsion droplets, nanocapsules, and coacervate droplets under various reaction conditions. These model protocells display distinct structural features with varied hydrophobic/hydrophilic feature, permeability and different efficiency to enrich biomolecules from surroundings and to promote catalytic reactions within the primitive compartments. The dynamic transitions between different supramolecular systems open a possible route for synthetic cells to utilize structural features to enrich biochemical species from surroundings and display distinct functions.

## 2 Results and Discussion

### 2.1 Protocell Transition via Interfacial DCC

**Figure 1:**
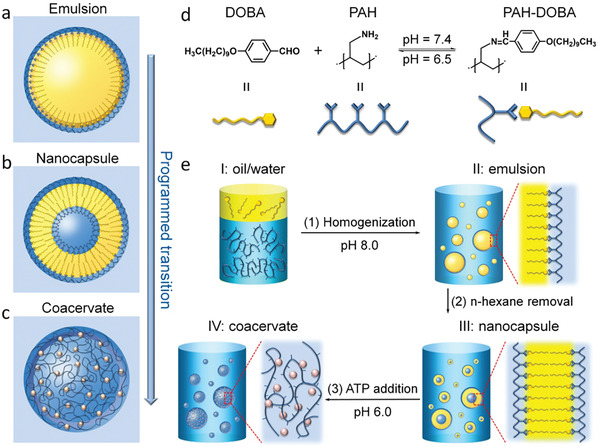
Directing protocell transition via modulating aldehyde‐amine dynamic covalent bonds. a–c) Structural transition of representative protocells including emulsion droplets, nanocapsules, and coacervate microdroplets. d) Reaction between an anionic polyelectrolyte (PAH) and a hydrophobic compound (DOBA) gave rise to a supra‐amphiphile (PAH‐DOBA) via DCC. e) The structural transition from biphasic oil/water system (Phase I) to emulsion via mechanical homogenization (Phase II), to capsules via solvent evaporation (Phase III), and to coacervate microdroplets after the addition of ATP under acid condition (Phase IV).

**Figure** **1** illustrates the construction of transitional protocells toward distinct compartments including emulsion droplets (Figure 1a), nanocapsules (Figure 1b), and coacervate microdroplets (Figure 1c) using poly(allylamine hydrochloride) (PAH) and 4‐decyloxybenzaldehyde (DOBA). Central to our approach is the dynamic bonding between the amine groups on PAH and the aldehyde group on DOBA through the Schiff reaction (Figure 1d).

Initially, PAH (10 mm) was dissolved in an aqueous solution while DOBA (10 mm) was dissolved in hexane (water/hexane, volume ratio of 4:1). Upon intense mechanical homogenization, the biphasic system (Phase I) transformed into an oil‐in‐water (o/w) emulsion (Phase II), which was stabilized by a brush‐like supra‐amphiphilic polymer bonded by dynamic imine bonds (PAH‐DOBA, as confirmed by NMR in Figure S1, Supporting Information). To verify the universality of this method, a fatty aldehyde (i.e., decanal) was used to form dynamic covalent bonds with PAH, and generated a brush‐like polymer PAH‐IMC_9_ (Figure S2, Supporting Information). In Phase III, we evaporated the organic solvent (i.e., hexane) in the amphiphilic polymer‐stabilized emulsion and obtained capsules in aqueous solution, in which the hydrophilic groups were exposed to the outer aqueous phase and the hydrophobic side chains were embedded to minimize the surface Gibbs energy. The imine bond is known to be sensitive to the acidic/basic condition, as such the nanocapsules were found to be thermodynamically stable under basic conditions (pH ≥ 7.4). As the pH lowered below 6.0, the breakage of imine bonds caused the regeneration of PAH and DOBA, as well as the disassembly of polymeric nanocapsules. At this stage, the addition of the negatively charged adenosine 5′‐triphosphate (ATP) can electrostatically complex with the polyelectrolyte PAH to generate membraneless coacervate microdroplets through liquid–liquid phase separation (Phase IV). In this way, we demonstrated the formation of synthetic compartments as protocell models that can transform in the presence of multiple stimuli (e.g., mechanical force, solvent evaporation, and acid/base equilibrium).

**Figure 2:**
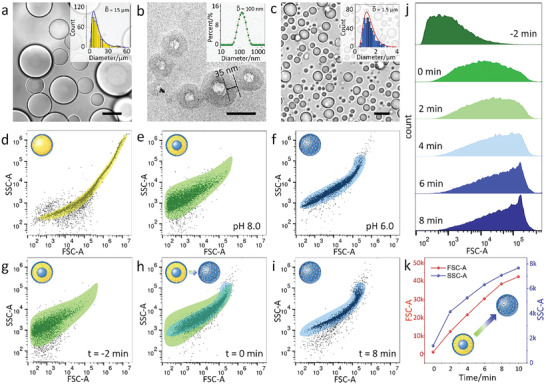
Characterizing the structural transitions of protocells. a) Optical microscopy image of the hexane‐in‐water emulsion, scale bar: 20 µm. b) Cryo‐TEM image of PAH‐DOBA nanocapsules, scale bar: 100 nm. c) Microscopy image of the PAH/ATP coacervates, scale bar: 2 µm. The insert images in (a–c) referred to the size distributions of the corresponding protocells. Dot plots of FSC versus SSC for individual protocell population of d) n‐hexane microdroplets, e) nanocapsules, and f) coacervate microdroplets, respectively. Flow cytometry plots of nanocapsules g) 2 min before the addition of ATP at pH 6.0, h) immediately after addition of ATP (at *t* = 0 min), and i) incubated for 8 min indicating the obliteration of nanocapsules and the formation of coacervate microdroplets. j) Time‐dependent flow cytometry during nanocapsule‐to‐coacervate transition. The increase in the FSC‐A signal indicated coacervation‐driven microdroplet formation. k) Time‐dependent medians of FSC‐A and SSC‐A values of the nanocapsule‐to‐coacervate transition process.

We further characterized the transition process of protocells with combined techniques. The in situ formation of o/w emulsion stabilized by brush polymer PAH‐DOBA was verified by the dye solubilization test. As shown in Figure S3, Supporting Information, the emulsion became foggy upon the addition of hydrophobic Sudan Black B (soluble in oil phase), while a continuous bluish color emerged when hydrophilic methyl blue was added (soluble in water phase). Meanwhile, the conductivity of the PAH‐DOBA stabilized o/w emulsion (17.43 µS cm^−1^) was measured to be lower than that in the PAH aqueous solution (206.0 µS cm^−1^), suggesting that amine groups on PAH were partially consumed by reacting with aldehyde. Optical microscopy image (**Figure** **2**a) confirmed the existence of emulsion droplets (≈15 µm) with high contrast edges.

Subsequent evaporation of hexane in the emulsion system resulted in emulsion‐to‐nanocapsule transition as evidenced by the change of macroscopic appearance from a turbid dispersion into an opalescent solution. Cryogenic transmission electron microscopy (Cryo‐TEM) and dynamic light scattering confirmed the formation of spherical nanocapsules with a diameter of 80–120 nm and membrane thickness of 15–40 nm (Figure 2b and Figure S4, Supporting Information). Moreover, as we removed the solvents (both water and hexane), and rehydrated the remaining solid, spherical nanocapsules formed again with a similar size of ≈130 nm (Figure S5, Supporting Information), indicating that nanocapsules were the thermodynamically favored morphology and could be produced from different pathways.

When the pH was adjusted to 6.0 and ATP was added, we observed a different type of protocell compartment known as coacervate microdroplets (average diameter < 1 µm). The emergence of microdroplets was believed to originate from PAH/ATP complexation driven by electrostatic attraction (Figure S6, Supporting Information), which caused liquid–liquid phase separation. Such coacervate droplets have been used as synthetic protocells that mimic the dynamical organization of membrane‐free organelles in living systems. Upon formation, these microdroplets grew fast in size largely due to colloidal coalescence and Ostwald ripening.^[^ ^39^ ^]^ As shown in microscopy (Figure 2c, Figure S7, and Movie S1, Supporting Information), the droplet size increases continuously from 0.8 µm at 125 s, to 1.3 µm at 250 s, to 1.8 µm at 375 s, and to 2.1 µm at 500 s. Moreover, the addition of ATP to PAH prior to DOBA was found to inhibit the formation of w/o emulsion (Figure S8, Supporting Information), possibly because PAH/ATP complexation suppressed covalent bonding between PAH and DOBA.

We performed flow cytometry to characterize the transition of emulsion droplets, nanocapsules and coacervates. As shown in Figure 2d–f, the individual populations of three protocells showed distinguishable 2D dot profiles of forward‐scattered (FSC) versus side‐scattered (SSC) light. Although all of the plots displayed a wide range of FSC and SSC values due to the polydispersity, the FSC and SSC ranged of emulsion droplets were broader than those of coacervates and nanocapsules, consistent with the results from optical microscopy measurements. The intensity of FSC light is known to be an important parameter to reflect the size of protocells. The polymer‐stabilized emulsion droplets showed an FSC median (165 000) higher than those of coacervates (9000) and nanocapsules (1170), indicating the larger size of emulsion and coacervate droplets. Meanwhile, the SSC values reflected the granularity and complexity of the protocells, where coacervates (3910) is higher than emulsion droplets (2770) and nanocapsules (1520). The high SSC for coacervates was believed arising from polyelectrolyte/ATP condensation into molecularly crowding droplets with enhanced internal complexity. The population of hollow nanocapsules showed the lowest SSC value because of their small size and the lack of complexity inside the nanocapsule cavity.

Figure 2g showed the dot plots immediately after the pH was lowered to 6.0, which gave similar pattern to that of the individual nanocapsule population observed in Figure 2e. The introduction of ATP did not cause noticeable changes in the dot plot (Figure 2h, *t* = 0 min), revealing a fast transition from nanocapsules to PAH/ATP coacervates within 8 min. The 2D dot plot at 8 min (Figure 2i) exhibited similar feature of the individual coacervate population (Figure 2f). We collected the FSC histograms every 2 min during the nanocapsule‐to‐coacervate transition (Figure 2j) and found a gradual increase in the mean FSC value, highlighting the continuous increase of the protocell sizes from nanocapsules (mean diameter ≈100 nm) to coacervates (mean diameter ≈1.5 µm) (Figure 2k).

### 2.2 Internal Structural Arrangement of Protocells

**Figure 3:**
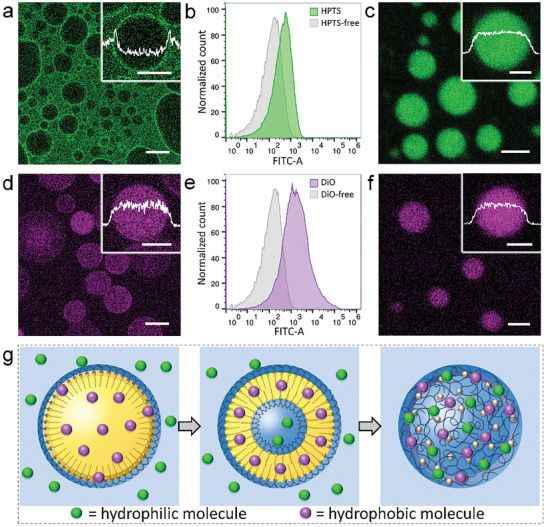
Molecular sequestration and redistribution in the structural transition protocells. The sequestration of a–c) HPTS and d–f) DiO in three types of protocells was examined by fluorescence microscopy a,c,d,f) and flow cytometry b,e). CLSM images of emulsion with excluded hydrophilic HPTS (a) and sequestered hydrophobic DiO (d). The FITC‐A histograms of nanocapsules the encapsulation of both hydrophilic HPTS (green peak, b) and hydrophobic DiO (purple peak, e), where the sequestration efficiency of DiO was higher than HPTS. Fluorescence microscopy images showing both HPTS (c) and DiO (f) were sequestrated in the coacervate microdroplets. Scale bars: 20 µm (a,d) and 2 µm (c,f). The inset fluorescence intensity profiles presented strong DiO fluorescence in the emulsion droplets and coacervate droplets, scale bars: 10 µm (a,d insets) and 1 µm (c,f insets). g) Schematic of structural transition of model protocells, where hydrophobic and hydrophilic payloads could be selectively located within different compartments.

We further explored the internal structure of protocells and their molecular sequestration property in different phases. As shown in **Figure** **3**a,d, the PAH‐DOBA emulsion droplets solubilized double‐chained 3,3′‐dioctadecyloxacarbocyanine perchlorate (DiO) but excluded 8‐hydroxypyrene‐1,3,6‐trisulfonic acid trisodium (HPTS), suggesting that the droplets were hydrophobic domain that can encapsulate DiO while the outer continuous surroundings were hydrophilic phase that sequestrated HPTS. Upon solvent evaporation, as shown in Figures 1 and 2, the emulsion droplets turned into nanocapsules. Unlike the emulsion droplets that can discriminate hydrophobic DiO and hydrophilic HPTS, the nanocapsules can uptake both fluorophores as can be seen from flow cytometry (Figure 3b,e), DiO or HPTS‐loaded nanocapsules were found to exhibit much stronger fluorescence. We hypothesized that DiO incorporated in the nanocapsule membrane while HPTS were embedded in the inner aqueous cavity. As shown in Figure 3c,f, upon lowering solution pH and the addition of ATP, the nanocapsules transformed into membraneless coacervate microdroplets via liquid–liquid phase separation. These cell‐sized droplets were observed to uptake both DiO and HPTS in the liquid phase. This is interesting since the previously reported coacervates formed from charged polyelectrolytes such as polylysine, DNA/RNA, and PDDA, selectively sequestrated hydrophilic dyes, proteins, and nucleic acids. The uptake of DiO in coacervate microdroplets were ascribed to the existence of hydrophobic domains due to the remaining DOBA hydrocarbon chains on PAH. Figures S9 and S10, Supporting Information, showed a systematic study of how these protocell models sequestrated a range of molecules. Unexpectedly, hydrophilic FTIC‐dextran, Hoechst and Rh6G located at the aqueous phase of o/w emulsion, while hydrophobic Nile Red was uptake by the emulsion droplets. TAMRA‐ssDNA was an exception that preferentially adsorbed at the oil/water interface, possibly due to the charge attraction between DNA and PAH‐DOBA. Similarly, all the compounds regardless of their molecular weight and charges could be encapsulated by capsules and coacervate droplets.

Taken together, we proposed that the structural transition of the protocells was accompanied by the relocation of hydrophilic/hydrophobic domains, resulting in varied molecular sequestration properties. Hydrophilic molecules (e.g., HPTS and DNA) experienced an external‐to‐internal sequestration along with the redistribution of the hydrophilic phase. Meanwhile, those hydrophobic payloads (e.g., DiO and Nile Red) remained inside the protocell such as emulsion droplets, nanocapsule membrane and coacervate droplets, even after enormous morphological transition (Figure 3g). In this way, it became possible for these two kinds of molecular payloads, originally separated from each other due to different hydrophobicity, to mix and react toward the formation of desired products in the same compartment.

### 2.3 Molecular Sequestration and Catalytic Reactions within Structural Transition Protocells

**Figure 4:**
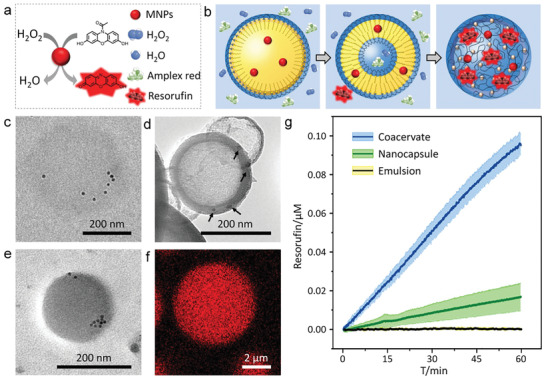
Nanozyme sequestration and catalytic reactions within protocells at different stages. a) Conversion of Amplex red into fluorescent resorufin by H_2_O_2_‐mediated oxidation catalyzed by MNP nanozyme. b) Hydrophobic MNPs embedded within three types of synthetic protocells generated by a programed transition. TEM images showing c) MNP‐encapsulated emulsion droplet, d) MNP‐embedded nanocapsules (black arrows indicated MNPs) and e) MNP‐encapsulated coacervate microdroplet. f) CLSM image of a coacervate microdroplet showing the red fluorescence of resorufin after catalytic reaction for 60 min. g) The increase in resorufin concentration for the emulsion droplets (black line), nanocapsules (green line) and coacervate microdroplets (blue line). Catalytic oxidation of Amplex red was initiated with the addition of H_2_O_2_. Error curves indicated the standard deviations in three replicating measurements.

Next, we explored the possibility to utilize the structural transition discussed above to transfer and relocate different biological components such as catalytic enzymes and substrates within the protocells. Toward that end, we used magnetic nanoparticles (MNPs) as an artificial enzyme. Coated with oleic acid, Fe_3_O_4_ nanoparticles were known to exhibit peroxidase‐like activity.^[^ ^40^ ^]^ These nanozymes were able to catalyze H_2_O_2_‐mediated oxidation of Amplex red generating resorufin that emitted red fluorescence (**Figure** **4**a). In this regard, our strategy was to sequester MNPs in the oil phase and Amplex red in the aqueous phase, respectively. After the transformation of the emulsion into nanocapsules, the nanozyme would reside in the hydrophobic domains, while the substrates (viz., Amplex red) were distributed in the aqueous phases outside and inside the nanocapsules. Finally, the nanozymes and substrates came together upon the formation of coacervates, generating red fluorescence from resorufin produced in the coacervates (Figure 4b).

We synthesized oleic‐acid‐coated MNPs following the thermal decomposition method (Figure S11, Supporting Information).^[^ ^41^ ^]^ These hydrophobic particles had a uniform diameter of 8 nm and could be readily dispersed in n‐hexane droplets without aggregation. Since the MNPs were on the nanometer scale in size, we used TEM to track their spatial distributions. Figure 4c presented a TEM image of the MNPs within n‐hexane droplets. After solvent evaporation, the emulsion was transformed into nanocapsules with MNPs embedding into the nanocapsule membranes or on the surface (Figure 4d). Finally, the MNPs were sequestered upon the formation of coacervate microdroplets (Figure 4e). The nanozymes could retain their activities within the three types of protocells through the whole transition process. With the addition of Amplex red during the process, only coacervate microdroplets showed red fluorescence after 60 min, which confirmed that the oxidation reaction took place within PAH/ATP microdroplets rather than in the emulsion or nanocapsules (Figure 4f).

We analyzed the MNP‐catalyzed oxidation of non‐fluorescent Amplex red to produce fluorescent resorufin in emulsion droplets, nanocapsules and coacervate microdroplets, respectively. To quantitatively determine the amount of enzymatic products, calibration curves of fluorescence intensity versus resorufin concentration in emulsion, capsules and coacervates were plotted (Figure S12, Supporting Information). Time‐dependent fluorescence intensities from the reaction product resorufin was then converted into resorufin concentration. As shown in Figure 4g, the resorufin concentration remained unchanged in the emulsion over 60 min, indicating the absence of H_2_O_2_ and Amplex red in MNP‐containing n‐hexane droplets. Apparently, the macroscopic segregation of the oil and aqueous phases prevented the encounter between the substrates and enzyme (Figure 4g, black curve). After the removal of n‐hexane leading to the formation of nanocapsule, MNPs were attached to or integrated into the nanocapsule membranes, while Amplex red could also be encapsulated in the aqueous lumen of nanocapsules. The drastic increase of the interfacial area promoted MNP‐Amplex red interactions and was responsible for the steady generation of resorufin. The enhanced MNP‐Amplex red interactions were also manifested in the kinetic curve during the testing time (green curve). As expected, the concentration of resorufin increased most rapidly after the sequestration of both MNPs and substrate molecules within coacervate microdroplet (blue curve). In a control experiment, the coacervates were disassembled by adding sodium chloride to eliminate the co‐localization effect of MNPs and Amplex red, where the catalytic reaction rate was found to significantly decrease (Figure S13, Supporting Information).

## 3 Conclusion

In this work, we presented a supramolecular system to establish a structural transition of protocells that were triggered by simple physiochemical cues such as mechanical force, solvent evaporation, acid/base equilibrium, and small molecules. By taking advantage of the adaptive bonding between the hydrophilic polyelectrolyte backbone and the hydrophobic group, we realized the protocell transition from a simple biphasic system, to o/w emulsion, nanocapsules, and coacervate droplets in this supramolecular system. In particular, by shifting the equilibrium of a dynamic chemical bond, we showed the compartments at different stages exhibited distinct internal molecular arrangement, interface permeability, molecular sequestration, and catalytic reaction efficiency. Our results highlight the possibility of using supramolecular systems to design synthetic protocells with functional and morphological adaptation, and shed light on the potential of constructing dynamic functional materials with supramolecular chemistry.

## 4 Experimental Section

### The Preparation of Emulsion

Typically, 200 µL of DOBA in n‐hexane (40 mm) and 800 µL of PAH in water (10 mm, pH = 8.0) were added into an Eppendorf centrifuge tube. The hexane‐in‐water emulsion was got with homogenizing at 3000 rpm by a homogenizer (IKA T10 basic ULTRA‐TURRAX) for 3 min at room temperature via the interfacial dynamic covalent reaction. Normally, the reaction took place for ≈1 h. The emulsion obtained in this way was stable for at least 12 h without demulsifying.

### Transition from Emulsion to Nanocapsules

The n‐hexane was removed with diaphragm pump (WELCH, MPC301Z) in a vacuum dryer (PC‐150, Shanghai Yueci Electronic Technology Co., Ltd.) at room temperature for ≈10 h. The internal pressure of the vacuum dryer was gradually reduced to 1.0 kg cm^−2^, and the emulsion was simultaneously stirred with a magnetic stirrer to help demulsify until it became completely transparent. Afterward, the solution was homogenized at 3000 rpm for 3 min to promote the PAH‐DOBA assembly into nanocapsules.

### Transition from Nanocapsules to Coacervates

The pH of nanocapsule dispersion was adjusted to 6.0 to achieve the release of PAH thus disassembly of nanocapsules. Then ATP (10 mm, pH = 6.0) was added to form PAH/ATP coacervate micro‐droplets at a monomer molar ratio of 2:1.

### Flow Cytometry Analysis

Dispersions of emulsion, nanocapsules, and coacervate micro‐droplets prepared by the above methods were investigated using an ACEA Novocyte flow cytometer. The flow speed was set at 8 µL min^−1^, and the 2D dot plots were determined for a total of 50 000 events. All measurements were performed on a Novo Cyte 2060R flow cytometer, and data analysis was performed with FlowJo 10.4 software.

### Cryo‐TEM

A small amount of PAH‐DOBA nanocapsule dispersion was applied to a holey carbon film (2 µm hole diameter) covered 300 mesh grids (R2/1 batch of GiG, CN), which was hydrophilized by plasma process in advance. The supernatant fluid was sucked up with LEICA EM GP (LEICA, DE) for 2.5 s, and then the film was quickly put into liquid ethane at −165 °C. The frozen sample was transferred into JEM‐2011 TEM using a Gatan 626 cryo‐transfer holder (Gatan, USA) and observed with an accelerating voltage of 120 kV.

### MNPs Mediated Peroxidase Activity in Three Types of Protocells

Before Schiff reaction, MNPs dispersed in n‐hexane were added into the DOBA solution to a final concentration of 0.1 mg mL^−1^, while Amplex red was put into PAH solution to a final concentration of 3.3 µm. The emulsion, nanocapsule, and coacervate dispersions were prepared using these solutions. Typically, 100 µL of emulsion, nanocapsule, or coacervate dispersion was put into a 96‐well plate, then added 3 µL of H_2_O_2_ (250 µm) and time‐dependent changes in red fluorescent intensity were recorded using a plate reader (CLARIO star plus, BMG Labtech) at excitation and emission wavelengths of 535 ± 30 and 590 ± 40 nm, respectively.

### Statistical Analysis

For diameter distributions of coacervate and emulsion droplet, more than 400 samples were analyzed. For the statistic of membrane thickness of nanocapsule, more than 60 samples were considered. The FITC‐A histogram and PerCP‐A histogram were normalized using following equation: $\mathrm{Normalized}\;\mathrm{count}\;=\frac{\mathrm{Count}(\mathrm{FI})}{\mathrm{Count}(\mathrm{FImax})}\;\times \;100$, where Count(FI) was the count of corresponding fluorescence intensity, and Count(FImax) was the peak value of the count. The calibration curves of fluorescence intensity versus resorufin concentration were obtained by linear fitting using Origin 2018 software. Corresponding fitting plots: Fluorescence intensity (coacervate system) = 5.74 × 10^5^ [Resorufin] + 420 (*R* ^2^ = 0.9982), Fluorescence intensity (emulsion system) = 3.06 × 10^5^ [Resorufin] − 3.57 (*R* ^2^ = 0.9985), Fluorescence intensity (nanocapsule system) = 1.13 × 10^6^ [Resorufin] + 900 (*R* ^2^ = 0.9981). All of error curves indicated the standard deviations in three replicating measurements.

## Conflict of Interest

The authors declare no conflict of interest.

## Supporting information

Supporting Information

Supplemental Movie 1

## Data Availability

The data that support the findings of this study are available from the corresponding author upon reasonable request.
