# Supplementary material for: Directing Transition of Synthetic Protocell Models via Physicochemical Cues‐Triggered Interfacial Dynamic Covalent Chemistry
Source: Adv Sci (Weinh). 2021 Jul 28;8(18):2101187. doi: 10.1002/advs.202101187 (PMC8456217; doi:10.1002/advs.202101187)
Supplement: Supplementary file 1 — Supporting Information [file ADVS-8-2101187-s001.pdf]

© 2021 Wiley-VCH GmbH

ADVANCED SCIENCE
Open Access

Supporting Information

for *Adv. Sci.*, DOI: 10.1002/advs.202101187

Directing Transition of Synthetic Protocell Models *via* Physicochemical Cues-Triggered Interfacial Dynamic Covalent Chemistry

*Yanglimin Ji, Wenjing Mu, Hua Wu and Yan Qiao**

Supporting Information

Directing Transition of Synthetic Protocell Models *via* Physicochemical Cues-Triggered Interfacial Dynamic Covalent Chemistry

*Yanglimin Ji, Wenjing Mu, Hua Wu and Yan Qiao**

Y. Ji, W. Mu, H Wu, Prof. Y. Qiao
Beijing National Laboratory for Molecular Sciences (BNLMS)
Laboratory of Polymer Physics and Chemistry
CAS Research/Education Center for Excellence in Molecular Sciences
Institute of Chemistry, Chinese Academy of Sciences
Beijing 100190, China
E-mail: yanqiao@iccas.ac.cn

Y. Ji, W. Mu, H Wu, Prof. Y. Qiao
University of Chinese Academy of Sciences
Beijing 100049, China

Supplementary Movie 1. Optical microscopy movie showing nanocapsule-to-coacervate transition shown at ×35 of real-time speed at 7 frames per second. Total recording time was 500 s.

Materials and methods

Materials. Poly(allylamine hydrochloride) (PAH, Mw = 17,000, 20 wt. % in H_2O, Sigma-Aldrich), 4-n-decyloxybenzaldehyde (DOBA, TCI) and adenosine 5′-triphosphate disodium salt hydrate (ATP, Sigma-Aldrich), Sudan Black B (Aladdin), methyl blue (Sinopharm Chemical Reagent Co., Ltd.), 8-hydroxypyrene-1,3,6-trisulfonic acid trisodium salt (HPTS, Sigma), rhodamine 6G (Rh6G, Sigma), Hoechst 33258 (Hoechst, Sigma), fluorescein isothiocyanate-dextran (FITC-dextran, Sigma, Mw = 70,000, FITC : glucose = 1 : 250), 3,3′-dioctadecyloxacarbocyanine perchlorate (DiO, Sigma), Nile red (Acros Organics), carboxytetramethylrhodamine-tagged single-stranded DNA oligonucleotides (TAMRA-ssDNA, Integrated DNA Technologies Inc., Belgium), peroxidase from horseradish (HRP,

Sigma), Amplex red (Sigma-Aldrich), resorufin (Bidepharm, 95%), iron chloride ($FeCl_3 \cdot 6H_2O$, Sigma-Aldrich, 98%, chunks), sodium oleate (TCI, 95%), 1-octadecene (Sigma, ≥95.0% (GC)) and oleic acid (Sigma, 95%) were used without further purification. Other chemicals were purchased from Beijing Chemical Plant Co., Ltd. All aqueous solutions were prepared with deionized water (18.2 MΩ·cm) produced by a Milli-Q System (Millipore, USA).

^{1}H nuclear magnetic resonance (^{1}H NMR) characterization. ^{1}H NMR spectra were recorded on a 300 MHz Bruker Fourier. The PAH-DOBA was prepared with the same procedure of emulsion, and the solvents were replaced with DMSO-d_6 and heated to 60 °C to ensure the complete dissolution.

Optical and fluorescent microscopy. The emulsion and coacervates were visualized using a confocal laser scanning microscopy (CLSM, Zeiss LSM880, Germany) with a Diode laser (405 nm for Hoechst), an Argon laser (488 nm for FITC, HPTS and DiO, 514 nm for Rh6G) and a HeNe543 laser (543 nm for Nile red, TAMRA). Detection bands were set at 500 - 550 nm for FITC and HPTS, 493 - 578 nm for DiO, 440 - 540 nm for Hoechst, 548 - 704 nm for TAMRA, 521 - 699 nm for Rh6G and 571 - 742 nm for Nile red. Generally, the fluorescent dyes were added to the emulsion and coacervate dispersion with a final concentration of 0.1 μM. Image analysis was performed with Image J software.

Dynamic light scattering (DLS) analysis. 1 mL of sample was filtered by needle type membrane filter (JIN TENG, diameter of 25 mm, pore diameter of 0.45 μm, PES), and the DLS measurements were obtained on Malvern Zetasizer Nano ZS. The refractive index and material refractive index were set as 1.33 and 1.454, respectively. Each sample was tested for at least three times.

Transmission electron microscopy (TEM). Typically, a droplet of sample dispersion (5 μL) was applied to a carbon film-covered 200 mesh grids (T10044, Beijing Xinxing Bairui Technology Co. LTD). After removing the excess solution with a piece of filter paper, the sample was dried in air and visualized on a JEM-1011 TEM with an accelerating voltage of 100 kV.

Synthesis of magnetic nanoparticles (MNPs). Oleic acid coated Fe_3O_4 nanoparticles were prepared by a thermal decomposition method [Park, J.; An, K.; Hwang, Y.; Park, J.-G.; Noh, H.-J.; Kim, J.-Y.; Park, J.-H.; Hwang, N.-M. and Hyeon, T., Nature Materials 2004, 3, 891-895.]. To synthesize iron-oleate complex, 18.25 g sodium oleate and 5.4 g $FeCl_3 \cdot 6H_2O$ was dissolved in a mixing solvent composed of 30 mL H_2O, 40 mL ethanol and 50 mL cyclohexane. The mixed solution was heated to 60 ℃ and kept for 2 hours. When the reaction was completed, the upper organic phase containing iron-oleate complex was washed twice with 30 mL milli-Q water in a separatory funnel. After washing, cyclohexane was evaporated off to obtain a waxy iron-oleate solid complex.

To synthesize monodisperse iron oxide (magnetite) nanocrystals, 4 g of iron-oleate precursors synthesized as described above and 0.5 mL oleic acid were dissolved in 20 mL 1-octadecene at room temperature. The reaction mixture was firstly heated to 120 °C for 2 hours, then 300 °C for 30 min. The resulting solution was then cooled to room temperature, and 50 mL of ethanol was added to wash the as-prepared nanocrystals.

Supplementary Figures

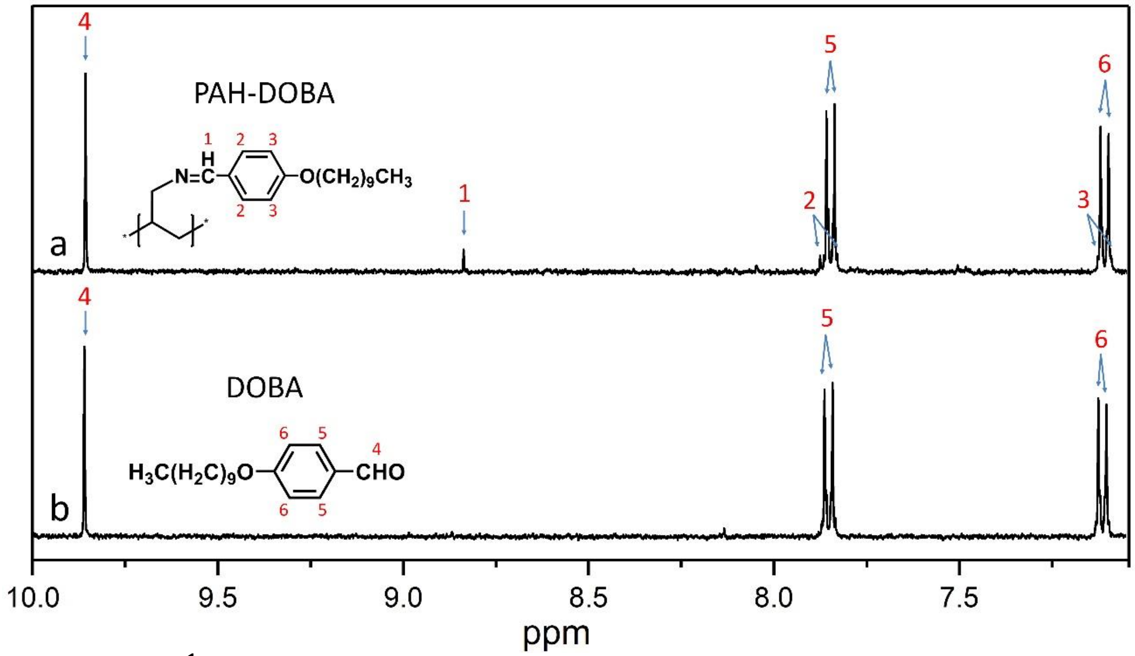

Figure S1. The ^{1}H NMR spectra of (a) the reactants, i.e. PAH and DOBA after Schiff reaction (product PAH-DOBA) and (b) the mixture of reactants without reaction. Peak 1 was assigned to imine hydrogen, which confirmed the formation of PAH-DOBA. The hydrogen atoms on the phenyl group of PAH-DOBA (Peak 2 and 3) overlapped with those on DOBA (Peak 5 and 6), and Peak 4 was assigned to aldehyde hydrogen. No NMR peak from PAH was observed from 7.0 to 10.0 ppm.

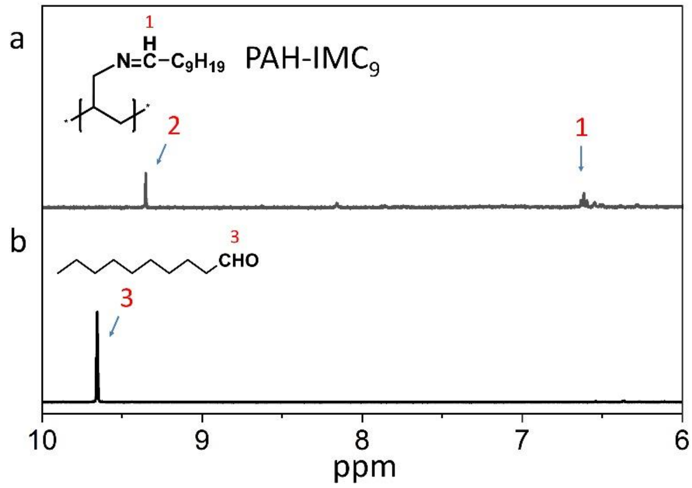

Figure S2. ^{1}H NMR spectra confirmed the formation of Schiff base from PAH and decanal: (a) PAH-IMC$_9$ and (b) decanal. The peak at 6.62 ppm assigned to imine hydrogen indicates the formation of PAH-IMC$_9$. The peak at 9.34 ppm is assigned to aldehyde hydrogen and was observed to shift to up-field relative to Peak 3 owning to the higher shielding effect caused by brush-like polymers.

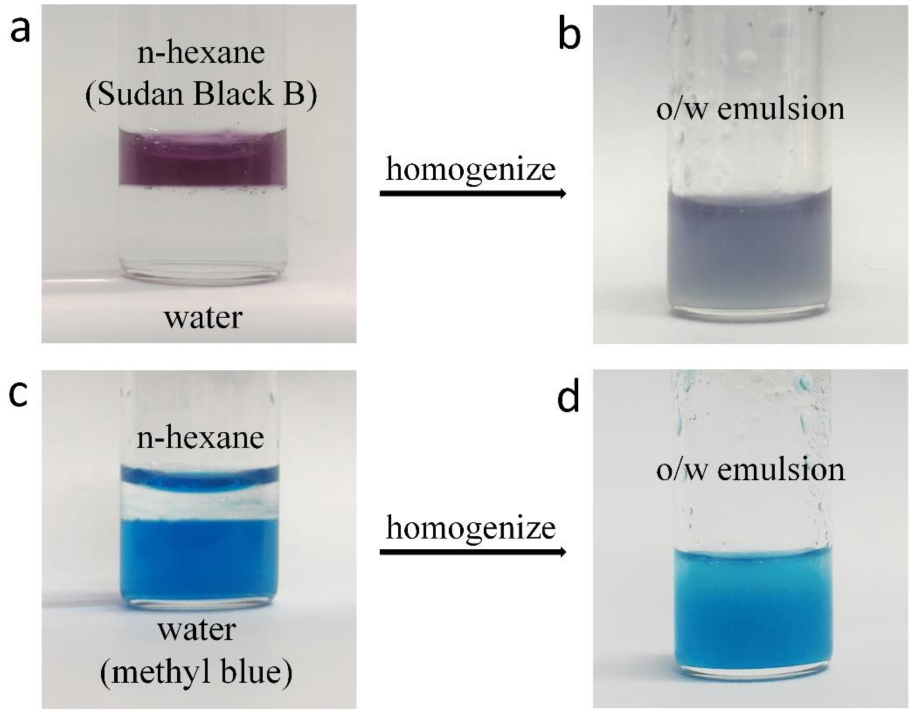

Figure S3. Phase transition from (**a,c**) **biphasic oil/water system to** (**b,d**) **the o/w emulsion.** (**a**) In the biphasic oil/water system, Sudan Black B was dissolved in n-hexane to give a purplish upper phase. (**b**) After homogenization, an o/w emulsion with the turbid foggy colour was formed. Sudan Black B is known to show a purple color in non-polar solvent and bluish black in lipid solution. The above colour change in (**a**,**b**) confirmed the formation of emulsion stabilized by PAH-DOBA. (**c**) A biphasic system containing methyl blue was in bottom aqueous phase and hexane in upper phase, and (**d**) the emulsion showed bluish declaring water was the continuous phase.

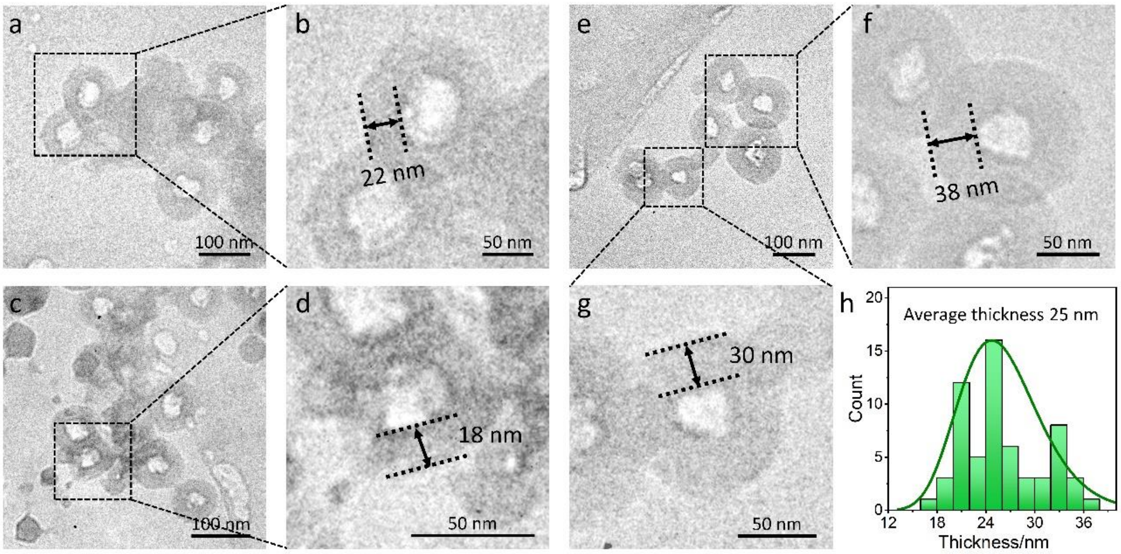

Figure S4. Cryo-TEM images of (a-g) PAH-DOBA nanocapsules and (h) the corresponding thickness distribution of capsule membrane. The thicknesses of the nanocapsule membranes ranged from 15 nm to 40 nm with an average value of 25 nm. Sixty nanocapsules were analyzed statistically.

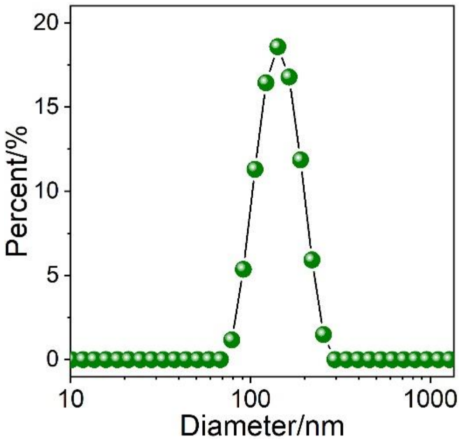

Figure S5. DLS data for the re-dispersed nanocapsules after the removal of solvent.

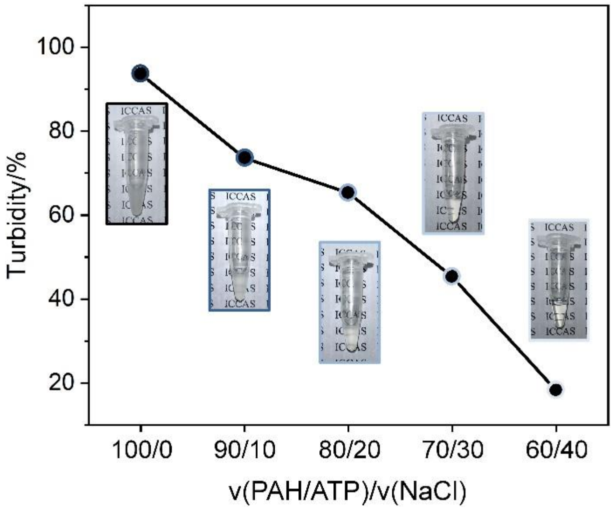

Figure S6. Disassembly of PAH/ATP coacervates with NaCl. The turbidity of coacervate dispersion decreased with the increase of volume fraction of NaCl. Inset images showed the turbid suspension turned into transparent solution.

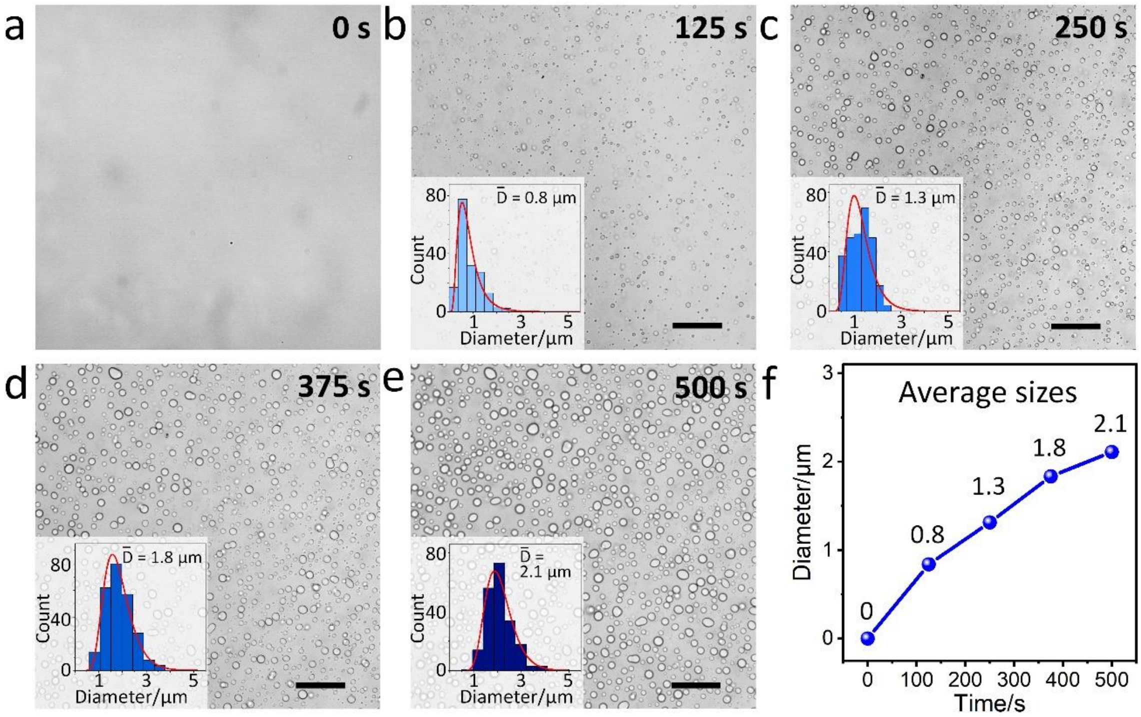

Figure S7. Time-dependent microscopy images showing the formation process of PAH/ATP coacervate microdroplets. (**a**) No coacervate microdroplets formed immediately after the addition of ATP solution. Coacervate microdroplets appeared after incubating for (**b**) 125 s, (**c**) 250 s, (**d**) 375 s and (**e**) 500 s, which further fused and grew bigger. Diameter distribution of coacervate droplets were shown in the insets of **b-e** based on statistical analysis of 200 samples, indicating a gradual growth of mean diameters over incubation time (**f**). Scale bars: 10 µm.

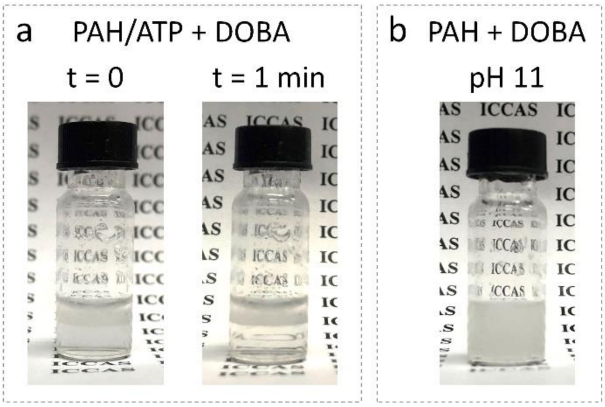

Figure S8. Effect of ATP on the oil/water system at pH 11. (**a**) Images of biphasic oil/water system containing PAH/ATP in water phase and DOBA in hexane. After homogenization, the mixture becomes turbid (t = 0 min) and turns into a biphasic system in 1 min. (**b**) A stable emulsion is obtained in the PAH/DOBA mixture after homogenization. The effect of ATP on the oil/water system (at pH 8 and 11) were studied based on the following experiments: At pH 8, PAH (20 mM, 200 μL) and ATP (20 mM, 200 μL) solution were mixed to form a turbid suspension of coacervates. At pH 11, PAH (20 mM, 200 μL) and ATP solution (20 mM, 200 μL) were mixed, where liquid-liquid phase separation didn't occur likely due to the absence of positive charges on PAH (the pKa of PAH is ~9.5). To this mixture, DOBA (20 mM in n-hexane, 200 μL) were added and the suspension was homogenized to facilitate the formation of emulsion through DCC reaction. The obtained dispersion demulsified in 1 min. As a control experiment, at pH 11, emulsion can form with PAH (10 mM, 400 μL) and DOBA (20 mM, 200 μL) by the method in our manuscript.

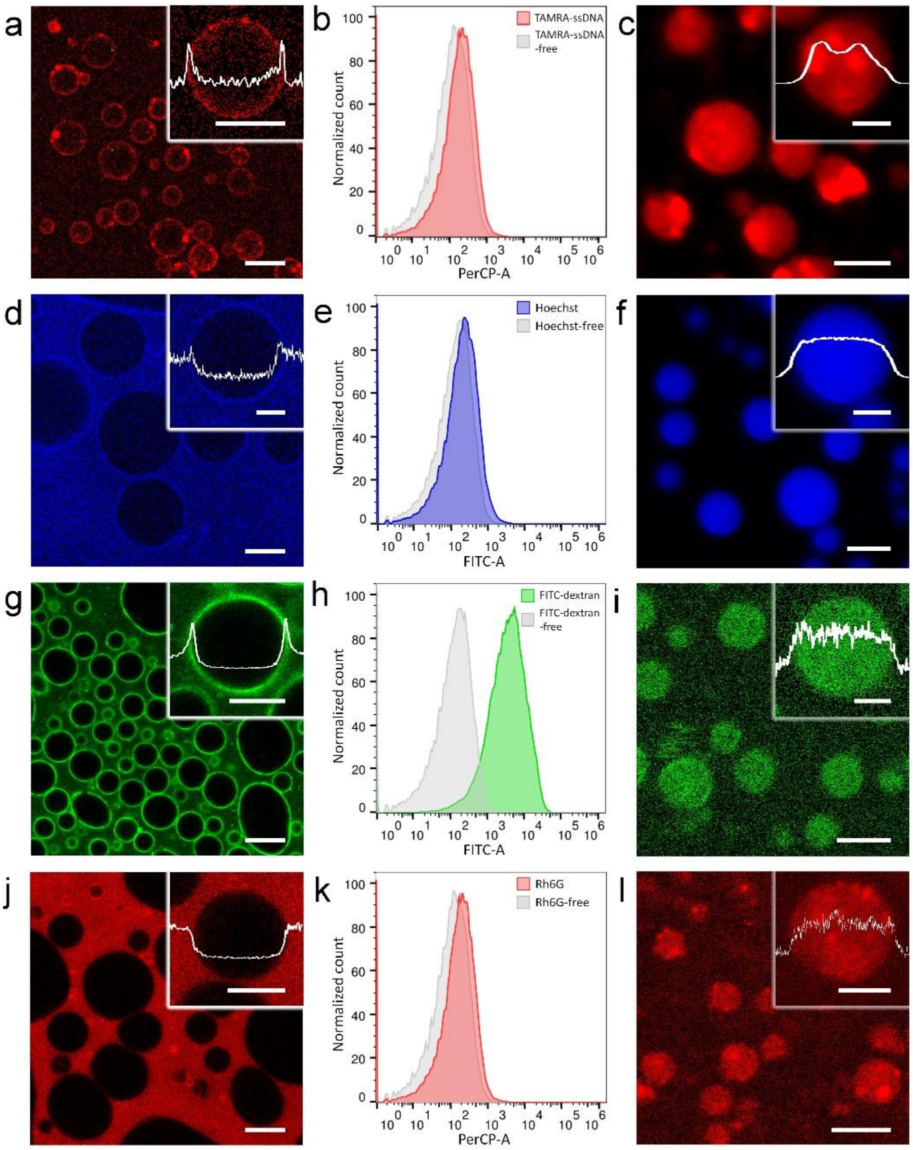

Figure S9. Sequestration of hydrophilic dye molecules in three types of protocells. The fluorescent images of emulsions showing negatively charged macromolecule TAMRA-ssDNA (**a**) absorbed at the oil/water interface of emulsion droplets, while neutral macromolecular FTIC-dextran (**g**), positively charged small molecular Hoechst (**d**) and Rh6G (**j**) were excluded by the emulsion microdroplets. It was also noted that Hoechst and FITC-dextran were enriched at the emulsion droplet interfaces. Flow cytometry histograms for nanocapsules showed slight increase in fluorescence intensities for TAMRA-ssDNA (**b**), Hoechst (**e**) and Rh6G (**k**). The fluorescence intensities were particularly high for FITC-dextran (**h**, 70 K). Fluorescence microscopy images for the TAMRA-ssDNA (**c**), Hoechst (**f**), FITC-dextran (**i**)

and Rh6G (**l**) were encapsulated within coacervates. Scale bars: 20 µm (**a**,**d**,**g**,**j**) and 2 µm (**c**,**f**,**i**,**l**). Insets showed the fluorescence intensity profiles across a single droplet, scale bars: 10 µm (**a**,**d**,**g**,**j** insets) and 1 µm (**c**,**f**,**i**,**l** insets).

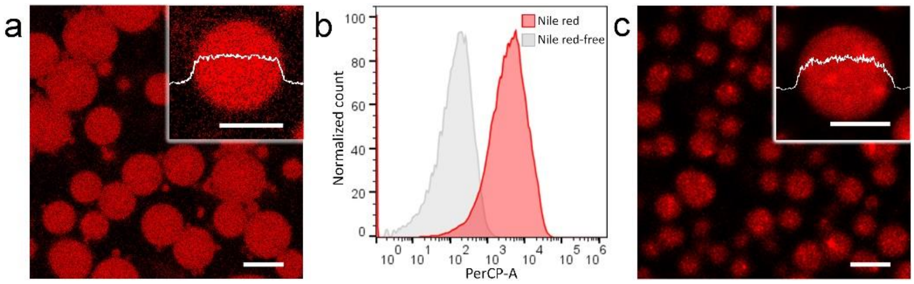

Figure S10. Sequestration of hydrophobic Nile red within protocells. (**a**) Fluorescence microscopy image of emulsion showed that Nile red was homogenously sequestered within n-hexane droplets, scale bar: 10 µm. (**b**) Histogram of PerCP-A for nanocapsules indicated high increase in fluorescence intensity after adding Nile red. (**c**) CLSM image of Nile red-doped PAH/ATP coacervates, scale bar: 2 µm. The insert images in (**a**,**c**) showed the fluorescence intensity profiles across single droplet, scale bars: 5 µm (**a** inset) and 1 µm (**c** inset).

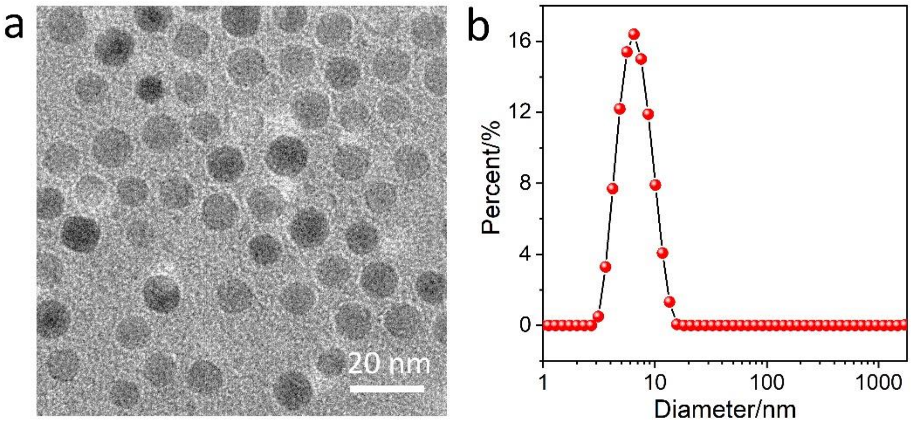

Figure S11. TEM and DLS characterization of MNPs. (**a**) TEM image of MNPs showed spherical particles with uniform size. (**b**) Size distribution by DLS showed an average MNP diameter of 8 nm.

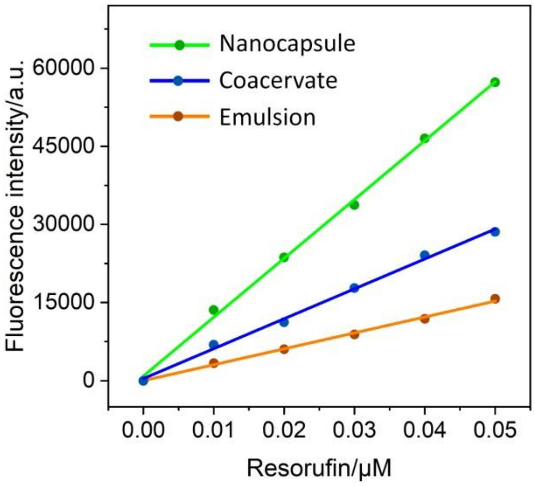

Figure S12. Calibration curves of fluorescence intensity vs. resorufin concentration.

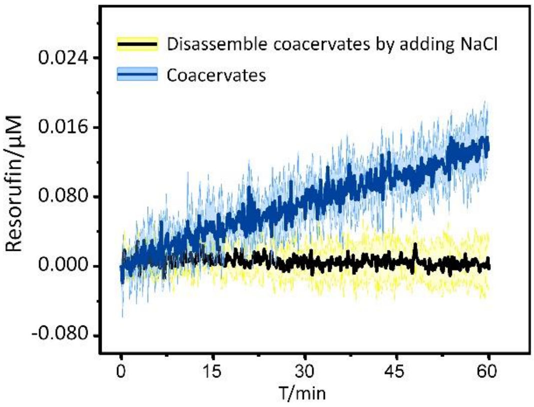

Figure S13. Effect of co-localization of MNPs and Amplex red on the catalytic activity of MNPs. The reaction was significantly decreased (black curve) when the PAH/ATP coacervates were disassembled with the addition of NaCl. Error curves indicate the standard deviations in three replicating measurements.
